# Fetal growth restriction in rural Bangladesh: a prospective study

**DOI:** 10.1186/s41182-018-0083-z

**Published:** 2018-02-06

**Authors:** Farzana Ferdous, Md. Harunor Rashid, Enbo Ma, Rubhana Raqib, Hiromi Hamada, Yukiko Wagatsuma

**Affiliations:** 1 Graduate School of Comprehensive Human Sciences, University of Tsukuba, 1-1-1 Tennodai, Tsukuba, Ibaraki 305-8575 Japan; 2 International Centre for Diarrhoeal Disease Research, Bangladesh (icddr,b), Dhaka, Bangladesh; 3 Department of Clinical Trial and Clinical Epidemiology, Faculty of Medicine, University of Tsukuba, 1-1-1 Tennodai, Tsukuba, Ibaraki 305-8575 Japan; 4 Health Promotion Center, Fukushima Medical University, Fukushima, Japan; 5 Department of Obstetrics and Gynecology, Tsukuba University Hospital, 1-1-1 Tennodai, Tsukuba, Ibaraki 305-8575 Japan

**Keywords:** Bangladesh, Fetal growth restriction (FGR), Gestational age, Low birth weight, Maternal malnutrition

## Abstract

**Background:**

Fetal growth restriction (FGR) and low birth weight(LBW) are serious public health problems. In developing countries, the incidence of low birth weight is predominantly the result of FGR, and both low birth weight and FGR are associated with neonatal death and later growth and development. Fetal growth charts are important for assessing the size of the fetus during pregnancy. The aims of this study were to describe the fetal growth pattern of a population in rural Bangladesh where maternal undernutrition is prevalent and to compare the timing of FGR in that population with WHO and INTERGROWTH- 21st international reference values.

**Methods:**

From November 2001 to October 2003, pregnant women were recruited in Matlab, a sub district of Bangladesh, and underwent three follow-up ultrasound examinations during pregnancy for measurement of the parameters of the fetal head, abdomen, and femur. The data were fitted to a linear-cubic model, and the derived values were compared with international reference values.

**Results:**

A total of 2678 singleton pregnancies were included in the analyses. The mean (SD) maternal age was 25.9 (5.8) years (range, 14–47 years). The mean (SD) early pregnancy BMI was 20.1 (2.6) kg/m^2^, and 27.6% of the women were underweight (BMI < 18.5 kg/m^2^). The growth of the biparietal diameter and abdominal circumference was significantly smaller throughout the pregnancy than the reference values (*P* ≤ 0.05). Moreover, a larger deviation in the growth of Bangladeshi fetuses was observed after 28 weeks of gestation when compared with the WHO and INTERGROWTH-21st reference fetal growth charts (*P* ≤ 0.05). After 28 weeks of gestation, the average Bangladesh estimated fetal weight gain per week of gestational age was significantly lower than the WHO estimated fetal weight by as much as 67.4 g (*P* ≤ 0.001).

**Conclusions:**

The present population-based study showed that fetuses were smaller in the third trimester when compared with the reference charts. Growth faltering started in the second trimester for all the biometric parameters for the head, abdomen, and femur. This finding provides more challenges concerning nutritional interventions.

**Electronic supplementary material:**

The online version of this article (10.1186/s41182-018-0083-z) contains supplementary material, which is available to authorized users.

## Background

Low birth weight (LBW) is a challenging public health problem in developing countries. Defined as weight at birth of less than 2500 g [1], LBW is a cause of infant mortality and impaired psychological development. Annually, nearly 20 million infants worldwide (15.5% of all births) are born with LBW; of those, 95.6% are from developing countries [2]. Indeed, 70% of all LBW babies are born in Asia, with more than a quarter of infants (27%) weighing less than 2500 g at birth in south-central Asia [2]. The incidence of LBW in Bangladesh is 21.6% [3], predominantly the result of fetal growth restriction (FGR), which is the highest in the world [4]. Moreover, that incidence of LBW is also a result of babies born small for gestational age (SGA) or preterm [5]. In general, FGR and SGA are considered interchangeably in studies, although differences exist [6]. FGR is growth considered as less than the normal growth potential of that specific infant, whereas SGA is usually considered as below the 10th percentile of a population-specific birth weight at exact gestational age [6]. During pregnancy, maternal undernutrition results in FGR and SGA, which are associated with an increased risk of perinatal morbidity and mortality [5, 7].

Former studies including a Bangladesh study reported that preterm infants whose growth had been restricted during pregnancy or born as SGA had a several-fold higher risk of neonatal death than did full-term infants [5, 8]. A study conducted in Sweden showed that small fetuses had a 10-fold higher risk of fetal death than did normal fetuses [9]. Another study showed that growth-restricted fetuses had a higher risk of stillbirth and that those who survived a compromised intrauterine environment were at increased risk for neonatal morbidity [10]. Therefore, early detection of FGR may help to reduce the associated morbidity and mortality [10]. Thus, measurements of fetal growth at different gestational ages are important for tracking the fetal size and can predict FGR in a majority of fetuses, which would allow appropriate prevention for fetuses at risk. Ultrasonographic measurements of the fetal biparietal diameter (BPD) and head circumference (HC) are used for assessing fetal growth and dating pregnancies [11]. Measurement of the fetal abdominal circumference (AC) is widely used as a parameter to estimate fetal size and weight [12]. Measurement of the femur length (FL) can be used to determine gestational age (GA) and fetal size as well as fetal abnormalities [13]. While numerous studies have been conducted to derive reference charts for fetal size, several of them had a suboptimal design, such as using hospital patients or having an inappropriately small sample size [14].

Fetal growth charts are used to identify any deviation from normal by plotting the measurements on charts. Such practices have proven to be effective in preventing adverse outcomes [15]. Most obstetrician and sonographers in developing countries follow fetal growth charts generated through studies on western populations with different socioeconomic and nutritional statuses from those of their own countries. Nevertheless, recent World Health Organization (WHO) and INTERGROWTH-21st studies have developed multiethnic international fetal growth charts to overcome the limits of existing reference charts for universal practices [11, 16]. However, the effectiveness and suitability for settings such as the Bangladesh setting have not yet been assessed, and Kiserud et al. themselves recommended that use of the WHO chart should be adjusted to the local context before being applied [11]. The authors also reported that WHO estimated fetal weight (EFW) and biometric measurements showed variation by countries in the reported charts, while India always fell at the bottom of the reference charts. On the other hand, INTERGROWTH-21st charts might be artificial for Bangladesh fetal growth because they were developed with the concept of optimal growth [16]. Hence, the feasibility of these international reference charts should be checked before being clinically used in Bangladesh.

Bangladesh is a country where the prevalence of maternal undernutrition and LBW are high; thus, it is important to understand the magnitude of the effect of maternal undernutrition at various timings of FGR. However, only a limited number of studies to develop fetal growth charts have been conducted in Bangladesh, and these used small numbers of participants and were conducted in tertiary-level hospitals; community-based studies have not been carried out. In addition, these studies neither compared the measurements with international reference values nor mentioned the timing of FGR. Given these deficiencies in the research as well as the importance of discussing the timing of deviations in fetal growth from a public health perspective to propose suitable nutrition interventions, the aims of this study were to develop fetal growth charts that describe the fetal growth pattern in a community of Bangladesh and to examine the timing of their deviations from international growth charts.

## Methods

The study was conducted in Matlab, a rural area of Bangladesh, where a Health and Demographic Surveillance System (HDSS) has been in operation since 1966. For the enrollment of the study participants, all women who tested positive for pregnancy by a urine pregnancy test during a 2-year period from November 2001 to October 2003 underwent ultrasound examinations. The present study was embedded into the maternal food and micronutrient supplementation study (the MINIMat study) (study registration: isrctn.org; identifier: ISRCTN16581394). The details of the study location and trial have been described elsewhere [17].

Pregnancy urine tests were offered to every woman who reported to the community health research workers (CHRWs) that her last menstrual period (LMP) was at least 2 weeks overdue or that she thought she was pregnant. The LMP date was determined by recall during the pregnancy identification interview at routine monthly household visits. If she tested positive for pregnancy, the woman was invited to join the study and the date of her LMP was recorded. She was invited to visit a nearby icddr,b clinic for evaluation of a viable fetus and estimation of the GA by ultrasound examination (conducted at 8–13 weeks’ GA). The inclusion criteria for a pregnant woman in the MINIMat study were that the fetus should be viable, the GA should be less than 14 weeks, and her consent to participate in the study was provided. The inclusion criteria for the present study in addition to the MINIMat study were that the pregnant woman should have an LMP date, the valid GA should have a limit of 21 days difference between the first trimester ultrasound-estimated GA and the LMP (if the first trimester ultrasound-estimated GA and LMP had a difference of more than 21 days, then we defined the LMP as erroneous), and she should have successfully completed three scheduled visits for fetal biometry measurements.

### Fetal biometry

All the enrolled women were examined by ultrasound during the clinic visits. The first ultrasound was conducted at enrollment at 8 to 13 weeks’ GA to measure the crown-rump length (CRL) to provide an ultrasound GA estimate. Otherwise, in some extreme situations such as larger fetuses (> 45 mm) or the head being visible, the GA was determined by the BPD. All the women were invited for further ultrasound examinations at around 14, 19, and 30 weeks of gestation. At each examination, three measurements were taken, and the examination took approximately 10 min.

Four ultrasound machines (SSA 320A, Justavision-200; Toshiba, Tokyo, Japan) with 3.5 MHz standard convex probes were used for the fetal biometry measurements. One ultrasound machine was placed in each clinic (a total of four clinics were used for the examinations), and measurements were performed according to the WHO ultrasound manual by nine trained paramedics over the two consecutive years in which the study participants were recruited [18]. The following four parameters were measured at the subsequent examinations: biparietal diameter (BPD), head circumference (HC), abdominal circumference (AC), and femur length (FL). The BPD was measured from the outer proximal skull (part nearest to the probe) to the inner distal skull (the part nearest to the probe). The HC was the ovoid measurement of the whole skull bones, where the image was at the level of the BPD and the measurement was taken by using the ellipse curve along the outer edge of the skull. The AC was measured at the level of the umbilical portion of the left portal vein by using the ellipse curve. The FL was measured from end to end with a full femoral image. The inter-observer and intra-observer variations and quality control of the ultrasound measurements have been described elsewhere [19]. The observed values were compared with the WHO and INTERGROWTH-21st international fetal biometry reference values for each parameter to determine the adequacy of the fetal growth for its GA [11, 16].

The women were formally invited to the clinic 1 week before they completed the scheduled numbers of weeks’ gestation based on the first trimester ultrasound-based LMP. The ultrasound-based LMP was used for scheduling of clinic visits since some women could not recall their LMP. The woman was asked whether she could attend; if she was unable, she was asked to attend either the week before or the week after, and if that was not possible, she was asked to visit the clinic at least before the next scheduled time. In this manner, while providing maximum opportunities for each woman to take the examinations, the study provided repeated longitudinal measurement data with 3 points (at 14 weeks [range, 11–20 weeks’ GA]; at 19 weeks [range, 15–28 weeks’ GA]; at 30 weeks [range, 24–41 weeks’ GA) for each woman spread throughout the gestational weeks.

Information on each woman’s age, parity, education, and household assets was collected from the surveillance system databases and from interviews with the women. Parity refers to the number of live or dead children delivered before the current pregnancy. Economic status was assessed by generating scores through principal component analysis based on household assets, housing structure, land occupation, and income. These scores were then indexed into quintiles, where 1 represents the poorest, and 5, the richest.

The height and weight of the pregnant women were measured at enrollment, at 6 to 13 weeks’ gestation. Weight was measured using electronic scales (Seca, Hamburg, Germany) with a precision of 100 g, and height, using locally made wooden scales with a precision of 0.1 cm. The body mass index (BMI) (kg/m^2^) was categorized into underweight (< 18.5), normal (18.5–< 25), and overweight (> 25). Birth weights were measured mostly within 72 h of birth by using SECA electronic scales (Seca), which are precise to 10 g. However, measurements were taken even if the newborns were reached after 72 h. The birth weight measurements taken within the first 24 h were used without adjustments. Measurements taken after 24 h to 30 days after birth were adjusted using an SD score transformation with the assumption that the birth weight tends to remain the same [4].

### Statistical analysis

The curves of the repeated measurements over the pregnancy period were fitted. The methodology of constructing the curves followed those from previous studies [20, 21]. The mean and standard deviation (SD) of each fetal parameter value were separately fitted to the polynomial regression model against its GA and generated the regression formulas [20, 21]. Data points beyond 6 SD from the regression line, fitted to the raw data, were unrealistic and were therefore removed. The best fitted polynomial curves were chosen by comparing the deviances and the goodness of fit of the model. The linear-cubic model was fitted to the mean of the raw data, and the linear model, to the SD. The standard deviation score (SDS) was fitted against the GA to assess the correctness of the model [20, 21]. The normal plot for the SDS was also checked to observe the correctness of the curve. The percentile curve was calculated according to the established formula [21]. The raw data were fitted to the 5th and 95th percentiles against the GA to assess the fitness of the curve [20, 21]. The linear models were performed to examine the line differences between the fitted and the reference lines throughout the gestation with or without the combination of the interaction effect (GA) on the main effect of the outcome variables (all 4 biological parameters) [20, 21]. This analysis demonstrated that the effect of levels of GA (explanatory variable) on the outcome variables was intrinsically tied to the specific level of two groups: the marginal contribution of GA is conditional on the groups (Bangladesh vs the reference). Therefore, an interaction effect of GA on the steady fetus growth between the present study and the reference charts was described as the combined effect of GA on fetus growth. To conduct this analysis, the present study used WHO and INTERGROWTH-21st reference fetal growth charts [11, 16]. Similarly to the WHO fetal growth study, the present study used Hadlock et al.’s third formula using the HC, AC, and FL to calculate the estimated fetal weight (EFW) [22]; thus, the EFW charts were compared with the WHO EFW charts only up to 35 weeks’ GA. For the EFW, the linear-power model was fitted to the mean and SD of the raw data. The proportion of small for gestational age (SGA) fetuses was also assessed and defined as an EFW below the 10th percentile for the GA. Although the present study data provided the values of each parameter from 13 weeks’ GA, the comparison charts were developed from 14 weeks’ GA owing to a lack of 13 weeks’ GA information in both the WHO and the INTERGROWTH-21st reference charts. Moreover, to determine the time of fetus growth faltering between the last two trimesters, we performed stratified analyses for each parameter. The *z* score was calculated to assess the deviation of the derived value from the expected reference value at each GA. The statistical analysis was performed using IBM SPSS (version 23.0; New York, NY, USA).

## Results

**Fig. 1:**
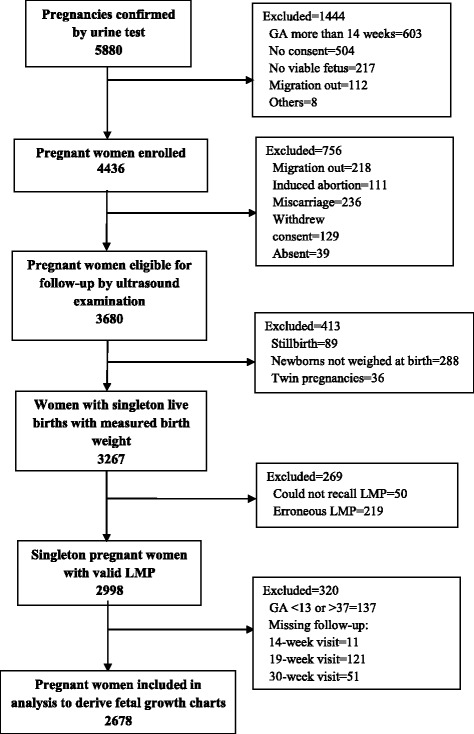
Flow chart of study participants. Abbreviation: GA gestational age, LMP last menstrual period

A total of 5880 women were identified as eligible for the MINIMat study. Of them, 1444 were excluded because of migration from the study area, refusal to participate, having a fetus whose GA exceeded the limits for the study or that was detected by ultrasound as being no longer viable, and other reasons (Fig. 1). A total of 4436 women were enrolled for the follow-up with ultrasound examinations; of them, 756 were excluded for reasons of migration, induced abortion, withdrawal of consent, spontaneous miscarriage, absence, and other reasons. A further 377 women were dropped from the study because the birth weight could not be measured (288 women) or the baby was stillborn (89). The remaining 3303 women had live-born infants. Of those infants, 36 were members of twin pairs and thus were excluded from the analysis of fetal growth, leaving 3267 newborns who contributed data.

A further 269 women had to be excluded owing to missing (could not recall) LMP dates (*n* = 50) or erroneous LMP information (*n* = 219). Additionally, 183 women who did not complete 3 scheduled visits were excluded from the analysis. Of the remaining 2813 women, 129 women with GA less than 13 weeks (falling into the first trimester, growth pattern different from the second and third), 8 women with GA more than 37 weeks (fetus numbers were fewer after 37 weeks’ GA) were excluded (Additional file 1: Table S1). Therefore, the finally valid 2678 singleton-birth women who had valid LMP dates and successfully completed the three scheduled visits were included in the analysis of fetal growth (Fig. 1).

The mean (SD) maternal age was 25.9 (5.8) years (range, 14–47 years). One third of the women (33.0%) were nulliparous. Only 68.3% of the women could read and write. The mean (SD) maternal height was 149.9 (5.3) cm. The mean (SD) early pregnancy BMI was 20.1 (2.6) kg/m^2^ and 27.6% of the women were underweight (Table 1). The average estimated birth weight was 2509.7 g at 37 weeks of birth GA, 2623.0 g at 38 weeks of birth GA, 2728.4 g at 39 weeks of birth GA, 2834.6 g at 40 weeks of birth GA, 2940.4 g at 41 weeks of birth GA, and 3044.4 g at 42 weeks of birth GA.

**Table 1:** Characteristics of study subjects (*n* = 2678)

Variables	*n* (%)
Maternal age (mean ± SD)	25.9 ± 5.8
Age group (year)	
14–19	405 (15.1)
20–24	779 (29.1)
25–29	769 (28.7)
30–34	492 (18.4)
≥ 35	233 (8.7)
Parity; *n* = 2669	
0	880 (33.0)
≥ 1	1789 (67.0)
Height (mean ± SD); *n* = 2676	149.9 ± 5.3
Educational status	
Illiterate	784 (29.3)
Can read only	64 (2.4)
Can read and write	1830 (68.3)
BMI, kg/m^2^ (mean ± SD); *n* = 2670	20.1 ± 2.6
BMI, kg/m^2^	
< 18.5	736 (27.6)
18.5–< 25	1786 (66.9)
≥ 25.0	148 (5.5)
Socioeconomic quintile	
1st (poorest)	513 (19.2)
2nd	525 (19.6)
3rd	541 (20.2)
4th	545 (20.4)
5th	554 (20.7)
Infant characteristics	
Birth weight (g) (mean ± SD)	2704.5 ± 402.0
Birth length (cm) (mean ± SD)	47.7 ± 2.1
Gestational age at birth (weeks) (mean ± SD)	38.8 ± 1.6

Abbreviation: *BMI* body mass index

The linear-cubic polynomial regression models were fitted to the mean, and the linear model to the SD with the raw data for all the biometric parameters (BPD, AC, HC, and FL), and the linear-power polynomial regression models were fitted to the mean and SD with the raw data for the EFW. The linear-cubic model and linear-power model gave a good fit to the data. The coefficients of determination (*R*^2^) were 0.966 for BPD, 0.968 for HC, 0.958 for AC, 0.968 for FL, and 0.967 for EFW (*P* < 0.001, respectively, indicating efficient correlations between all the biometric parameters and GA).

The fitted standardized residual of the SDS with the regression line against the GA showed that more than 90% of the observations lay within the fitted line for all the parameters. The normal plots of the SDS of each parameter appeared to be a linear pattern. For the data with fitted percentiles for each parameter, more than 90% of the values appeared to lie within the fitted line. The percentiles for each parameter were calculated on the basis of the established equation. Table 2 shows the fetal growth equations of the mean and SD derived for each parameter for this population. The fetal growth charts were developed on the basis of the fetal growth equation. The fitted values of the 1st, 2.5th, 5th, 10th, 25th, 50th, 75th, 90th, 95th, 97.5th, and 99th percentiles by the GA of the 4 parameters and EFW are given in the Additional file 1: Tables S2-S6.

**Table 2:** Regression formula used to generate ultrasound biometry charts and tables of biparietal diameter (BPD), head circumference (HC), abdominal circumference (AC), femur length (FL), and estimated fetal weight (EFW)

BPD	
Mean = − 24.624 + 3.739* GA - 0.0004972* GA3	
SD = 1.994 + 0.053* GA	
HC	
Mean = − 94.476 + 14.273* GA - 0.0020276* GA3	
SD = 8.182 + 0.121*GA	
AC	
Mean = − 78.497 + 11.708* GA - 0.0009802* GA3	
SD = 2.059 + 0.461* GA	
FL	
Mean = − 32.653 + 3.499* GA - 0.0005433* GA3	
SD = 1.975 + 0.028* GA	
EFW	
Mean = 0.0095699*GA**4.4937733	
SD = 0.00265083*GA**3.287346	

Abbreviation: *GA* gestational age, *SD* standard deviation

The fetal growth rate declined more in the third trimester than those of the two reference charts. This trend was obvious for the BPD and AC. The average growth per week of the BPD was 34.7 mm up to 20 weeks (WHO, 36.6 mm; INTERGROWTH-21st, 38.9 mm), then 60.8 mm from 21 to 29 weeks (WHO, 62.7 mm; INTERGROWTH-21st, 64.3 mm), and from 30 to 37 weeks, it was 81.7 mm (WHO, 83.9 mm; INTERGROWTH-21st, 86.4 mm). The growth of the AC was 110.0 mm per week up to 20 weeks (WHO, 114.9 mm; INTERGROWTH-21st, 114.3 mm), thereafter 198.4 mm from 21 to 29 weeks (WHO, 207.1 mm; INTERGROWTH-21st, 201.5 mm), and then 276.4 mm up to 37 weeks (WHO, 293.3 mm; INTERGROWTH-21st, 288.4 mm).

However, a slightly different growth pattern was observed for the HC and the FL. For the HC, up to 29 weeks, the growth rate was smaller, but in the last trimester, it became larger than those of both international references. The HC growth was 131.4 mm up to 20 weeks (WHO, 135.9 mm; INTERGROWTH-21st, 135.32 mm), and 229.7 mm from 21 to 29 weeks (WHO, 231.6 mm; INTERGROWTH-21st, 228.8 mm), and 306.4 mm up to 37 weeks (WHO, 305.0 mm; INTERGROWTH-21st, 303.3 mm). The FL growth was close to the WHO growth but slightly larger than the INTERGROWTH-21st growth (Additional file 1: Table S2-S6).

**Fig. 2:**
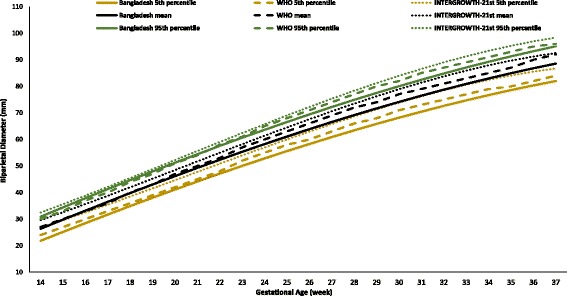
Comparison of biparietal diameter (BPD) with WHO [11] and INTERGROWTH-21st [16] values

**Fig. 3:**
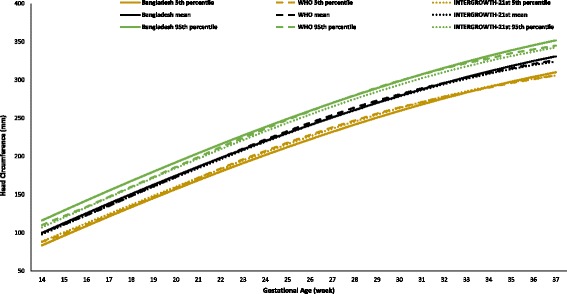
Comparison of head circumference (HC) with WHO [11] and INTERGROWTH-21st [16] values

**Fig. 4:**
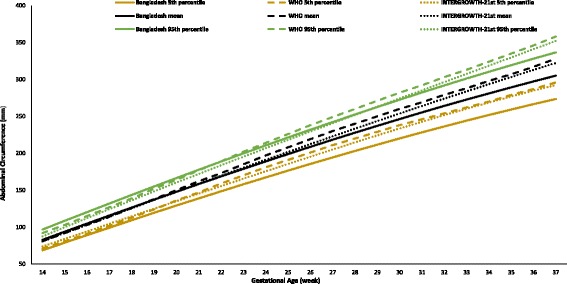
Comparison of abdominal circumference (AC) with WHO [11] and INTERGROWTH-21st [16] values

**Fig. 5:**
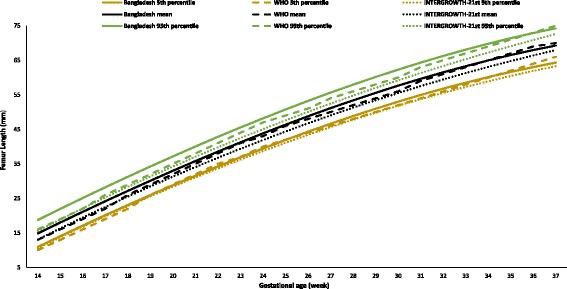
Comparison of femur length (FL) with WHO [11] and INTERGROWTH-21st [16] values

**Fig. 6:**
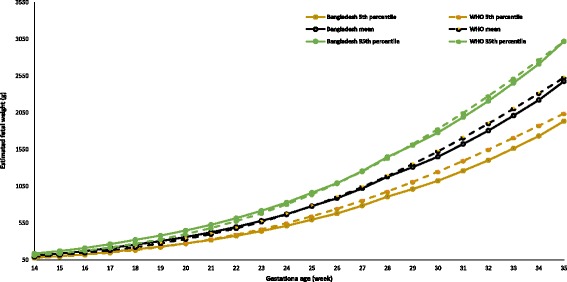
Comparison of estimated fetal weight (EFW) with WHO [11] reference values up to 35 weeks’ GA

Figures 2, 3, 4, 5, and 6 show the comparisons of each parameter derived from this study with the international reference values. Comparison of the difference between the fitted line and the reference chart mean line showed that only the BPD and AC were significantly smaller than the WHO and INTERGROWTH-21st international reference values (*P* < 0.05). The deviation of our derived values from the expected WHO and INTERGROWTH-21st international reference values at each GA showed that the mean BPD was consistently smaller than both the WHO (*β* = − 1.60, *P* = 0.005) and the INTERGROWTH-21st (*β* = − 3.61, *P* ≤ 0.001) reference curves throughout the observed period. However, up to 27 weeks the BPD was smaller than the WHO reference curve, where the GA had a combined effect on this linear growth (*β* = − 0.14, *P* < 0.001). The mean HC was larger than the WHO reference curve up to 27 weeks with the combined effect of the GA (*β* = 0.43, *P* < 0.001), but thereafter, the deviation become larger until 37 weeks when compared with the WHO reference value with the combined effect of the GA (*β* = 0.96, *P* < 0.001). For the INTERGROWTH-21st reference curve, the mean HC was larger than the reference curve up to 27 weeks without the combined effect of the GA (β = 1.76, *P* = 0.006), but thereafter, no deviation was observed up to 37 weeks. The mean AC was consistently smaller than the WHO (*β* = − 1.07, *P* < 0.001) and INTERGROWTH-21st (*β* = − 0.82, *P* < 0.001) reference curves throughout the pregnancy with the combined effect of the GA. The mean FL was significantly larger than the WHO reference curve up to 27 weeks (*β* = 1.14, *P* = 0.003; no combined effect of the GA), but thereafter, the growth started declining more than the WHO reference values with a combined effect of the GA (*β* = − 0.30, *P* < 0.001). On the other hand, the mean FL was significantly larger than the INTERGROWTH-21st reference curve throughout the pregnancy (*β* = 1.84, *P* < 0.001; no combined effect of the GA). The average Bangladesh EFW gain per week of GA was 14.6 g less than the WHO reference value; however, this difference was not significant (*P* ≥ 0.05). After 28 weeks of GA, the average Bangladesh EFW gain per week of GA was significantly lower than the WHO reference values by as much as 67.4 g (*P* ≤ 0.001). Nine percent of the fetuses were identified as SGA.

## Discussion

The results from the present study indicate the preparatory time of FGR and amount of deviation of Bangladesh fetuses in comparison with two recent international growth charts where measurements were taken longitudinally [11, 16]. The present study developed fetal growth charts for BPD, HC, AC, FL, and EFW and compared them with the WHO [11] and INTERGROWTH-21st [16] reference charts to determine the adequacy of the fetus growth of a rural community of Bangladesh. This study also found that our predicted equations from Bangladesh were similar to the WHO predicted fetal growth equations [11].

The deviation of the present study values from the reference values increased with increased gestational age. The growth of the derived values was smaller than the 50th percentiles of the reference values in the third trimester. Throughout the pregnancy, significantly smaller growth as compared with the mean reference values was observed for all the parameters other than for the FL, which was smaller with respect to the GA only from 30 weeks of gestation than the WHO and the INTERGROWTH-21st FL reference values. However, the mean HC value was larger for Bangladeshi fetuses throughout the period than both the WHO reference values and the INTERGROWTH-21st reference values. The reason might be fetal growth restriction at the last two trimesters of pregnancy of Bangladeshi fetuses and their dolichocephalic head size. Dolichocephalic head size is one of the outcomes of intrauterine constraints in which the occipitofrontal diameter (OFD) is larger than the BPD [23, 24]. However, the OFD values are lacking in the WHO reference data; thus, the present study did not compare these OFD values to give a strong justification for the larger mean HC of Bangladeshi fetuses. Moreover, the comparison with the international reference values showed that the growth faltering started after the 20th gestational week for all the fetal parameters and that faltering was gradually increased with increasing GA. The present study implies that up to 20 weeks, the fetal growth of Bangladeshi children might be adequate, although some of the parameter growth was deviated from the beginning of the second trimester, which indicates symmetric growth abnormalities of Bangladeshi fetuses. Symmetric growth inhibition arises during the 4th to 20th weeks of gestation, when fetal growth occurs primarily through cellular division and produces an undersized fetus with fewer cells of normal size that is characterized by a proportional lack of growth, including smaller dimensions of the head, abdomen, and femur [24].

The Bangladesh EFW in the last trimester of pregnancy was smaller than the WHO EFW values, which evidently shows the growth deviating time of Bangladeshi fetuses. The growth of all the parameters was smaller from 30 weeks of gestation in comparison with the growth up to 20 weeks of gestation. These findings imply that the growth impairment of fetuses in Bangladesh might start at the beginning of the second trimester and becomes apparent in the third trimester. Several factors may be responsible for the present study’s observed differences from the reference population. The differences are likely caused by different population characteristics [25]. The present study findings reflected the maternal pre-pregnancy and early pregnancy physical condition and markedly different maternal stature of Bangladeshi women, as they are lighter and shorter than western women. In the present study, the height of half of the women was below 150 cm, influencing a probability of multigenerational fetal growth restriction [26]. Maternal malnutrition might be another explanation for the growth faltering found in the present study. Both these factors of maternal malnutrition and maternal stature might affect fetal growth during pregnancy [27].

The maternal nutritional state both before pregnancy and after pregnancy has a significant effect on fetal development. In the present study, around one third of the study participants were undernourished, and previous studies stated that maternal undernutrition with different nutrient deficiencies in mid-pregnancy can reduce or increase placental weight [28]. Maternal nutrition factors associated with placental homeostasis influence fetal growth. Inadequate nutrition during pregnancy hampers its normal process and causes fetal growth restriction [7]. Maternal undernutrition status and overnutrition status reduce the placental-fetal blood flow and cause stunted fetuses. Impaired placental syntheses of nitric oxide and polyamines may provide an explanation for the growth restriction [29]. Thus, the present study recommends further study to be conducted on fetal growth, with consideration of maternal nutritional status and factors that are influential for fetal development.

The present study used the methods of previous studies [20, 21] to overcome the methodological weakness for fitting the curves. The distinguishing point of the present study was that fetuses that underwent three longitudinal measurements of all the variables were included for the construction of fetal growth charts. All steps in the statistical methods gave proper attention to the variability in the measurements that occur with increasing gestation and carefully assessed the goodness of fit of the models obtained [20].

The present study has several strengths. First, it included larger observations than did most other studies that developed fetal growth charts. Furthermore, it was a population-based study with longitudinal fetal growth measurement of pregnancies that used only singleton live births. The fetuses were followed from early fetal life and their growth was confirmed by 3 scheduled visits that were spread widely from 13 to 37 weeks of gestation, thus enabling us to create fetal growth charts. Finally, the present study compared its derived means and SDs with internationally published recommended reference charts.

The limitations to this study were that the measurement of the fetuses was not equally distributed at all gestational ages by scheduled visits and that the study was conducted at only 1 location in Bangladesh. The purposes of the present study were to describe the growth pattern of Bangladeshi fetuses in a rural population and to identify the time of growth faltering using reference charts. Therefore, the chart in this study should not be used as a reference for optimal growth in Bangladesh. A further limitation was the methodological differences for pregnancy dating between the present study and the WHO and INTERGROWTH-21st studies. Both these international studies accepted a difference of 7 days from the LMP [11, 16], while this study accepted a difference of 21 days. Further limitations were that this study could not construct hybrid fetal or birth weight charts nor did it provide any comparison birth weight charts at late pregnancy. Moreover, maternal morbidity information such as information on preeclampsia, diabetes, and fetal anomalies were not available to the authors.

## Conclusions

In the present study, we developed fetal growth charts for a Bangladeshi population. The fetal growth in the third trimester for all the parameters was smaller than those of the international reference values. Growth restriction for all the parameters started from the second trimester. Thus, the findings of the present study suggest that special attention is required to identify the critical time of fetal growth restriction so that appropriate nutrition intervention can be provided at the pre-pregnancy stage and at the early stage of pregnancy. In addition to their usefulness for the assessment of fetal size and growth, these findings suggest the importance of improving the health status of women of reproductive age in developing countries.

## Additional file

Additional file 1: **Table S1.** Distribution of gestational age (GA, week) at the 14, 19 and 30 weeks clinic visits, with LMP dates. **Table S2.** Growth chart for fetal biparietal diameter (BPD, mm) by percentile with SD. **Table S3.** Growth chart for fetal head circumference (HC, mm) by percentile with SD. **Table S4.** Growth chart for fetal abdominal circumference (AC, mm) by percentile with SD. **Table S5.** Growth chart for fetal femur length (FL, mm) by percentile with SD. **Table S6.** Growth chart for estimated fetal weight (g) by percentile with SD. (DOC 348 kb)

## Abbreviations

AC: Abdominal circumference
BMI: Body mass index
BPD: Biparietal diameter
CHRWs: Community health research workers
CRL: Crown-rump length
FGR: Fetal growth restriction
FL: Femur length
GA: Gestational age
HC: Head circumference
HDSS: Health and demographic surveillance system
LBW: Low birth weight
LMP: Last menstrual period
MINIMat Study: Maternal and Infant Nutrition Intervention Study in Matlab
OFD: Occipitofrontal diameter
SD: Standard deviation
SDS: Standard deviation score
SGA: Small for gestational age
