# Supplementary material for: Fetal growth restriction in rural Bangladesh: a prospective study
Source: Trop Med Health. 2018 Feb 6;46:3. doi: 10.1186/s41182-018-0083-z (PMC5801896; doi:10.1186/s41182-018-0083-z)
Supplement: Additional file 1: — Table S1. Distribution of gestational age (GA, week) at the 14, 19 and 30 weeks clinic visits, with LMP dates. Table S2. Growth chart for fetal biparietal diameter (BPD, mm) by percentile with SD. Table S3. Growth chart for fetal head circumference (HC, mm) by percentile with SD. Table S4. Growth chart for fetal abdominal circumference (AC, mm) by percentile with SD. Table S5. Growth chart for fetal femur length (FL, mm) by percentile with SD. Table S6. Growth chart for estimated fetal weight (g) by percentile with SD. (DOC 348 kb) [file 41182_2018_83_MOESM1_ESM.doc]

**Additional file 1**

**Table S1. Distribution of gestational age (GA, week) at the 14, 19 and 30 weeks clinical visits, with LMP dates.**

| GA by LMP, week | Eligible with LMP for observation n=2813 | 14-week visit, n (%) | 19-week visit, n (%) | 30-week visit, n (%) |
| --- | --- | --- | --- | --- |
| 11 | 18 | 18 (0.6) | - | - |
| 12 | 111 | 111 (3.9) | - | - |
| 13 | 399 | 399 (14.2) | - | - |
| 14 | 849 | 849 (30.2) | - | - |
| 15 | 773 | 769 (27.3) | 4 (0.1) | - |
| 16 | 466 | 444 (15.8) | 22 (0.8) | - |
| 17 | 266 | 172 (6.1) | 94 (3.3) | - |
| 18 | 380 | 38 (1.4) | 342 (12.2) | - |
| 19 | 746 | 11 (0.4) | 735 (26.1) | - |
| 20 | 748 | 2 (0.1) | 746 (26.5) | - |
| 21 | 485 | - | 485 (17.2) | - |
| 22 | 228 | - | 228 (8.1) | - |
| 23 | 97 | - | 97 (3.4) | - |
| 24 | 39 | - | 38 (1.4) | 1 (0.0004) |
| 25 | 13 | - | 12 (0.4) | 1 (0.0004) |
| 26 | 5 | - | 5 (0.2) | - |
| 27 | 29 | - | 4 (0.1) | 26 (0.9) |
| 28 | 101 | - | 1 (0.0004) | 100 (3.6) |
| 29 | 355 | - | - | 355 (12.6) |
| 30 | 832 | - | - | 832 (29.6) |
| 31 | 706 | - | - | 706 (25.1) |
| 32 | 445 | - | - | 445 (15.8) |
| 33 | 189 | - | - | 189 (6.7) |
| 34 | 82 | - | - | 82 (2.9) |
| 35 | 33 | - | - | 33 (1.2) |
| 36 | 23 | - | - | 23 (0.8) |
| 37 | 12 | - | - | 12 (0.4) |
| 38 | 4 | - | - | 4 (0.2) |
| 39 | 2 | - | - | 2 (0.1) |
| 40 | 1 | - | - | 1 (0.0004) |
| 41 | 1 | - | - | 1(0.0004) |
| Mean ± SD |  | 14.6 ± 1.3 | 19.9 ± 1.6 | 30.8 ± 1.6 |
| Total |  |  | | n=2813 |

**Table S2. Growth chart for fetal biparietal diameter (BPD, mm) by percentile** with SD

| Weeks  of gestation | No of Fetus (measurement) | 1^st^ | 2.5^th^ | 5^th^ | 10^th^ | 25^th^ | 50^th^ | 75^th^ | 90^th^ | 95^th^ | 97.5^th^ | 99^th^ | SD |
| --- | --- | --- | --- | --- | --- | --- | --- | --- | --- | --- | --- | --- | --- |
| 13 | 731 | 16.64 | 17.63 | 18.46 | 19.46 | 21.09 | 22.89 | 24.69 | 26.32 | 27.32 | 28.15 | 29.14 | 2.68 |
| 14 | 879 | 19.98 | 21.00 | 21.84 | 22.86 | 24.52 | 26.36 | 28.19 | 29.86 | 30.87 | 31.72 | 32.73 | 2.74 |
| 15 | 637 | 23.28 | 24.32 | 25.18 | 26.21 | 27.91 | 29.78 | 31.65 | 33.35 | 34.38 | 35.25 | 36.28 | 2.79 |
| 16 | 318 | 26.54 | 27.59 | 28.47 | 29.53 | 31.26 | 33.16 | 35.07 | 36.80 | 37.85 | 38.73 | 39.79 | 2.84 |
| 17 | 208 | 29.75 | 30.82 | 31.72 | 32.79 | 34.56 | 36.50 | 38.44 | 40.20 | 41.27 | 42.17 | 43.24 | 2.90 |
| 18 | 522 | 32.91 | 34.00 | 34.91 | 36.00 | 37.80 | 39.78 | 41.75 | 43.55 | 44.64 | 45.56 | 46.65 | 2.95 |
| 19 | 792 | 36.01 | 37.12 | 38.06 | 39.17 | 41.00 | 43.01 | 45.02 | 46.85 | 47.96 | 48.89 | 50.00 | 3.00 |
| 20 | 644 | 39.06 | 40.19 | 41.14 | 42.27 | 44.13 | 46.18 | 48.22 | 50.09 | 51.22 | 52.16 | 53.29 | 3.05 |
| 21 | 352 | 42.05 | 43.20 | 44.16 | 45.31 | 47.21 | 49.29 | 51.37 | 53.27 | 54.42 | 55.38 | 56.53 | 3.11 |
| 22 | 170 | 44.98 | 46.15 | 47.13 | 48.30 | 50.22 | 52.34 | 54.46 | 56.38 | 57.55 | 58.53 | 59.70 | 3.16 |
| 23 | 66 | 47.84 | 49.03 | 50.02 | 51.21 | 53.17 | 55.32 | 57.48 | 59.44 | 60.63 | 61.62 | 62.81 | 3.21 |
| 24 | 25 | 50.63 | 51.84 | 52.85 | 54.06 | 56.05 | 58.24 | 60.43 | 62.42 | 63.63 | 64.64 | 65.85 | 3.27 |
| 25 | 7 | 53.35 | 54.58 | 55.61 | 56.83 | 58.86 | 61.08 | 63.31 | 65.33 | 66.56 | 67.59 | 68.82 | 3.32 |
| 26 | 4 | 55.99 | 57.24 | 58.29 | 59.54 | 61.59 | 63.85 | 66.11 | 68.17 | 69.42 | 70.46 | 71.71 | 3.37 |
| 27 | 15 | 58.56 | 59.83 | 60.89 | 62.16 | 64.25 | 66.54 | 68.84 | 70.93 | 72.19 | 73.26 | 74.52 | 3.43 |
| 28 | 117 | 61.05 | 62.34 | 63.41 | 64.70 | 66.82 | 69.15 | 71.48 | 73.61 | 74.89 | 75.97 | 77.26 | 3.48 |
| 29 | 542 | 63.45 | 64.76 | 65.85 | 67.16 | 69.32 | 71.68 | 74.05 | 76.20 | 77.51 | 78.60 | 79.91 | 3.53 |
| 30 | 836 | 65.77 | 67.10 | 68.21 | 69.53 | 71.72 | 74.12 | 76.52 | 78.71 | 80.04 | 81.15 | 82.47 | 3.58 |
| 31 | 615 | 68.00 | 69.34 | 70.47 | 71.82 | 74.04 | 76.47 | 78.91 | 81.13 | 82.47 | 83.60 | 84.95 | 3.64 |
| 32 | 311 | 70.13 | 71.50 | 72.64 | 74.01 | 76.26 | 78.73 | 81.20 | 83.46 | 84.82 | 85.96 | 87.33 | 3.69 |
| 33 | 136 | 72.17 | 73.56 | 74.72 | 76.10 | 78.39 | 80.90 | 83.40 | 85.69 | 87.07 | 88.23 | 89.62 | 3.74 |
| 34 | 50 | 74.12 | 75.52 | 76.70 | 78.10 | 80.42 | 82.96 | 85.50 | 87.82 | 89.22 | 90.40 | 91.80 | 3.80 |
| 35 | 34 | 75.96 | 77.38 | 78.57 | 80.00 | 82.34 | 84.92 | 87.50 | 89.85 | 91.27 | 92.47 | 93.89 | 3.85 |
| 36 | 11 | 77.69 | 79.13 | 80.34 | 81.79 | 84.17 | 86.78 | 89.40 | 91.78 | 93.22 | 94.43 | 95.87 | 3.90 |
| 37 | 12 | 79.32 | 80.78 | 82.01 | 83.47 | 85.88 | 88.53 | 91.18 | 93.60 | 95.06 | 96.29 | 97.75 | 3.96 |
| Total | 8034 |  |  |  |  |  |  |  |  |  |  |  |  |

**Table S3. Growth chart for fetal head circumference (HC, mm) by percentile with SD**

| Weeks  of gestation | No of fetus (measurement) | 1^st^ | 2.5^th^ | 5^th^ | 10^th^ | 25^th^ | 50^th^ | 75^th^ | 90^th^ | 95^th^ | 97.5^th^ | 99^th^ | SD |
| --- | --- | --- | --- | --- | --- | --- | --- | --- | --- | --- | --- | --- | --- |
| 13 | 731 | 63.89 | 67.50 | 70.52 | 74.13 | 80.08 | 86.62 | 93.15 | 99.10 | 102.71 | 105.74 | 109.35 | 9.76 |
| 14 | 879 | 76.77 | 80.43 | 83.49 | 87.14 | 93.17 | 99.78 | 106.40 | 112.42 | 116.08 | 119.14 | 122.79 | 9.88 |
| 15 | 637 | 89.48 | 93.18 | 96.28 | 99.98 | 106.08 | 112.78 | 119.47 | 125.57 | 129.27 | 132.37 | 136.07 | 10.00 |
| 16 | 318 | 102.01 | 105.76 | 108.89 | 112.64 | 118.81 | 125.59 | 132.37 | 138.54 | 142.28 | 145.42 | 149.16 | 10.12 |
| 17 | 208 | 114.35 | 118.14 | 121.31 | 125.10 | 131.34 | 138.20 | 145.06 | 151.31 | 155.10 | 158.27 | 162.06 | 10.24 |
| 18 | 522 | 126.47 | 130.31 | 133.52 | 137.35 | 143.67 | 150.61 | 157.55 | 163.87 | 167.71 | 170.92 | 174.75 | 10.36 |
| 19 | 792 | 138.38 | 142.26 | 145.51 | 149.39 | 155.78 | 162.80 | 169.83 | 176.22 | 180.10 | 183.35 | 187.22 | 10.48 |
| 20 | 644 | 150.06 | 153.98 | 157.27 | 161.19 | 167.66 | 174.76 | 181.87 | 188.33 | 192.26 | 195.54 | 199.47 | 10.60 |
| 21 | 352 | 161.49 | 165.46 | 168.79 | 172.75 | 179.30 | 186.48 | 193.66 | 200.20 | 204.17 | 207.50 | 211.46 | 10.72 |
| 22 | 170 | 172.67 | 176.69 | 180.05 | 184.06 | 190.67 | 197.94 | 205.21 | 211.82 | 215.83 | 219.19 | 223.21 | 10.84 |
| 23 | 66 | 183.58 | 187.64 | 191.04 | 195.10 | 201.79 | 209.13 | 216.48 | 223.17 | 227.23 | 230.62 | 234.68 | 10.97 |
| 24 | 25 | 194.22 | 198.32 | 201.75 | 205.86 | 212.62 | 220.05 | 227.47 | 234.24 | 238.34 | 241.78 | 245.88 | 11.09 |
| 25 | 7 | 204.56 | 208.70 | 212.18 | 216.32 | 223.16 | 230.67 | 238.18 | 245.01 | 249.16 | 252.63 | 256.78 | 11.21 |
| 26 | 4 | 214.59 | 218.78 | 222.29 | 226.49 | 233.40 | 240.98 | 248.57 | 255.48 | 259.68 | 263.19 | 267.38 | 11.33 |
| 27 | 15 | 224.31 | 228.55 | 232.09 | 236.33 | 243.31 | 250.99 | 258.66 | 265.64 | 269.88 | 273.43 | 277.66 | 11.45 |
| 28 | 117 | 233.70 | 237.98 | 241.57 | 245.85 | 252.91 | 260.66 | 268.41 | 275.47 | 279.75 | 283.34 | 287.62 | 11.57 |
| 29 | 542 | 242.75 | 247.08 | 250.70 | 255.03 | 262.16 | 269.99 | 277.82 | 284.95 | 289.28 | 292.90 | 297.23 | 11.69 |
| 30 | 836 | 251.45 | 255.82 | 259.48 | 263.85 | 271.05 | 278.97 | 286.88 | 294.09 | 298.46 | 302.12 | 306.49 | 11.81 |
| 31 | 615 | 259.78 | 264.19 | 267.89 | 272.31 | 279.59 | 287.58 | 295.58 | 302.86 | 307.27 | 310.97 | 315.39 | 11.93 |
| 32 | 311 | 267.73 | 272.19 | 275.93 | 280.39 | 287.74 | 295.82 | 303.90 | 311.25 | 315.71 | 319.45 | 323.91 | 12.05 |
| 33 | 136 | 275.30 | 279.80 | 283.58 | 288.08 | 295.51 | 303.67 | 311.82 | 319.25 | 323.76 | 327.53 | 332.03 | 12.18 |
| 34 | 50 | 282.46 | 287.01 | 290.82 | 295.37 | 302.87 | 311.11 | 319.35 | 326.85 | 331.40 | 335.21 | 339.76 | 12.30 |
| 35 | 34 | 289.21 | 293.81 | 297.66 | 302.25 | 309.83 | 318.15 | 326.47 | 334.04 | 338.63 | 342.48 | 347.08 | 12.42 |
| 36 | 11 | 295.54 | 300.18 | 304.06 | 308.70 | 316.35 | 324.75 | 333.15 | 340.80 | 345.44 | 349.33 | 353.97 | 12.54 |
| 37 | 12 | 301.43 | 306.11 | 310.03 | 314.72 | 322.44 | 330.92 | 339.40 | 347.12 | 351.81 | 355.73 | 360.42 | 12.66 |
| Total | 8034 |  |  |  |  |  |  |  |  |  |  |  |  |

**Table S4. Growth chart for fetal abdominal circumference (AC, mm) by percentiles with SD**

| Weeks  of gestation | No of fetus (measurement) | 1^st^ | 2.5^th^ | 5^th^ | 10^th^ | 25^th^ | 50^th^ | 75^th^ | 90^th^ | 95^th^ | 97.5^th^ | 99^th^ | SD |
| --- | --- | --- | --- | --- | --- | --- | --- | --- | --- | --- | --- | --- | --- |
| 13 | 731 | 52.79 | 55.77 | 58.27 | 61.25 | 66.16 | 71.55 | 76.95 | 81.86 | 84.84 | 87.34 | 90.31 | 8.05 |
| 14 | 879 | 62.89 | 66.04 | 68.68 | 71.83 | 77.02 | 82.73 | 88.43 | 93.62 | 96.77 | 99.41 | 102.56 | 8.51 |
| 15 | 637 | 72.91 | 76.23 | 79.01 | 82.33 | 87.80 | 93.81 | 99.83 | 105.30 | 108.62 | 111.40 | 114.72 | 8.97 |
| 16 | 318 | 82.83 | 86.32 | 89.25 | 92.74 | 98.49 | 104.82 | 111.14 | 116.89 | 120.38 | 123.31 | 126.80 | 9.44 |
| 17 | 208 | 92.67 | 96.33 | 99.39 | 103.06 | 109.09 | 115.72 | 122.35 | 128.39 | 132.05 | 135.12 | 138.78 | 9.90 |
| 18 | 522 | 102.40 | 106.23 | 109.44 | 113.27 | 119.59 | 126.53 | 133.47 | 139.79 | 143.62 | 146.83 | 150.66 | 10.36 |
| 19 | 792 | 112.03 | 116.03 | 119.38 | 123.38 | 129.98 | 137.23 | 144.48 | 151.08 | 155.08 | 158.44 | 162.44 | 10.82 |
| 20 | 644 | 121.54 | 125.71 | 129.21 | 133.38 | 140.26 | 147.82 | 155.38 | 162.26 | 166.43 | 169.93 | 174.10 | 11.28 |
| 21 | 352 | 130.94 | 135.28 | 138.92 | 143.27 | 150.43 | 158.29 | 166.16 | 173.32 | 177.66 | 181.30 | 185.65 | 11.74 |
| 22 | 170 | 140.21 | 144.73 | 148.51 | 153.02 | 160.47 | 168.64 | 176.82 | 184.26 | 188.77 | 192.56 | 197.07 | 12.20 |
| 23 | 66 | 149.36 | 154.04 | 157.97 | 162.65 | 170.38 | 178.86 | 187.34 | 195.07 | 199.75 | 203.68 | 208.36 | 12.66 |
| 24 | 25 | 158.37 | 163.22 | 167.29 | 172.15 | 180.15 | 188.94 | 197.74 | 205.74 | 210.60 | 214.67 | 219.52 | 13.12 |
| 25 | 7 | 167.24 | 172.26 | 176.47 | 181.50 | 189.79 | 198.89 | 207.99 | 216.27 | 221.30 | 225.51 | 230.54 | 13.58 |
| 26 | 4 | 175.96 | 181.15 | 185.51 | 190.71 | 199.27 | 208.68 | 218.09 | 226.66 | 231.86 | 236.21 | 241.41 | 14.05 |
| 27 | 15 | 184.53 | 189.89 | 194.39 | 199.76 | 208.61 | 218.33 | 228.04 | 236.89 | 242.26 | 246.76 | 252.12 | 14.51 |
| 28 | 117 | 192.94 | 198.47 | 203.11 | 208.65 | 217.78 | 227.81 | 237.84 | 246.97 | 252.51 | 257.15 | 262.68 | 14.97 |
| 29 | 542 | 201.18 | 206.89 | 211.67 | 217.38 | 226.79 | 237.13 | 247.47 | 256.88 | 262.59 | 267.37 | 273.08 | 15.43 |
| 30 | 836 | 209.26 | 215.14 | 220.06 | 225.94 | 235.63 | 246.28 | 256.92 | 266.62 | 272.49 | 277.42 | 283.30 | 15.89 |
| 31 | 615 | 217.15 | 223.20 | 228.27 | 234.32 | 244.30 | 255.25 | 266.20 | 276.18 | 282.23 | 287.30 | 293.35 | 16.35 |
| 32 | 311 | 224.87 | 231.09 | 236.30 | 242.52 | 252.78 | 264.04 | 275.30 | 285.56 | 291.78 | 296.99 | 303.21 | 16.81 |
| 33 | 136 | 232.40 | 238.79 | 244.14 | 250.53 | 261.07 | 272.64 | 284.21 | 294.75 | 301.14 | 306.49 | 312.89 | 17.27 |
| 34 | 50 | 239.73 | 246.29 | 251.79 | 258.35 | 269.17 | 281.05 | 292.93 | 303.75 | 310.31 | 315.81 | 322.37 | 17.73 |
| 35 | 34 | 246.86 | 253.60 | 259.24 | 265.97 | 277.07 | 289.26 | 301.45 | 312.55 | 319.28 | 324.92 | 331.65 | 18.19 |
| 36 | 11 | 253.79 | 260.70 | 266.48 | 273.38 | 284.76 | 297.26 | 309.76 | 321.14 | 328.04 | 333.82 | 340.72 | 18.66 |
| 37 | 12 | 260.51 | 267.58 | 273.51 | 280.58 | 292.24 | 305.05 | 317.86 | 329.52 | 336.59 | 342.52 | 349.59 | 19.12 |
| Total | 8034 |  |  |  |  |  |  |  |  |  |  |  |  |

**Table S5. Growth chart for fetal femur length (FL, mm) by percentile with SD**

| Weeks  of gestation | No of fetus (measurement) | 1^st^ | 2.5^th^ | 5^th^ | 10^th^ | 25^th^ | 50^th^ | 75^th^ | 90^th^ | 95^th^ | 97.5^th^ | 99^th^ | SD |
| --- | --- | --- | --- | --- | --- | --- | --- | --- | --- | --- | --- | --- | --- |
| 13 | 731 | 6.19 | 7.06 | 7.78 | 8.65 | 10.07 | 11.64 | 13.21 | 14.63 | 15.50 | 16.22 | 17.09 | 2.34 |
| 14 | 879 | 9.33 | 10.20 | 10.94 | 11.81 | 13.26 | 14.84 | 16.43 | 17.87 | 18.75 | 19.48 | 20.36 | 2.37 |
| 15 | 637 | 12.42 | 13.30 | 14.05 | 14.93 | 16.39 | 18.00 | 19.60 | 21.06 | 21.95 | 22.69 | 23.58 | 2.40 |
| 16 | 318 | 15.46 | 16.36 | 17.11 | 18.00 | 19.48 | 21.11 | 22.73 | 24.21 | 25.10 | 25.85 | 26.75 | 2.42 |
| 17 | 208 | 18.45 | 19.36 | 20.12 | 21.02 | 22.52 | 24.16 | 25.80 | 27.30 | 28.20 | 28.96 | 29.87 | 2.45 |
| 18 | 522 | 21.38 | 22.30 | 23.07 | 23.99 | 25.50 | 27.16 | 28.82 | 30.33 | 31.25 | 32.02 | 32.94 | 2.48 |
| 19 | 792 | 24.26 | 25.19 | 25.97 | 26.89 | 28.42 | 30.10 | 31.78 | 33.31 | 34.24 | 35.02 | 35.94 | 2.51 |
| 20 | 644 | 27.07 | 28.01 | 28.80 | 29.74 | 31.28 | 32.98 | 34.68 | 36.23 | 37.16 | 37.95 | 38.89 | 2.54 |
| 21 | 352 | 29.82 | 30.77 | 31.57 | 32.51 | 34.08 | 35.79 | 37.51 | 39.08 | 40.02 | 40.82 | 41.77 | 2.56 |
| 22 | 170 | 32.50 | 33.46 | 34.26 | 35.22 | 36.80 | 38.54 | 40.28 | 41.86 | 42.82 | 43.62 | 44.58 | 2.59 |
| 23 | 66 | 35.11 | 36.08 | 36.89 | 37.86 | 39.46 | 41.21 | 42.97 | 44.57 | 45.54 | 46.35 | 47.32 | 2.62 |
| 24 | 25 | 37.64 | 38.62 | 39.44 | 40.42 | 42.04 | 43.81 | 45.59 | 47.20 | 48.18 | 49.00 | 49.98 | 2.65 |
| 25 | 7 | 40.10 | 41.09 | 41.92 | 42.91 | 44.54 | 46.33 | 48.13 | 49.76 | 50.75 | 51.58 | 52.57 | 2.68 |
| 26 | 4 | 42.47 | 43.47 | 44.31 | 45.31 | 46.96 | 48.77 | 50.58 | 52.23 | 53.23 | 54.07 | 55.07 | 2.70 |
| 27 | 15 | 44.76 | 45.77 | 46.62 | 47.63 | 49.30 | 51.13 | 52.96 | 54.62 | 55.63 | 56.48 | 57.49 | 2.73 |
| 28 | 117 | 46.96 | 47.98 | 48.84 | 49.86 | 51.54 | 53.39 | 55.24 | 56.92 | 57.94 | 58.80 | 59.82 | 2.76 |
| 29 | 542 | 49.07 | 50.10 | 50.97 | 52.00 | 53.70 | 55.57 | 57.43 | 59.13 | 60.17 | 61.03 | 62.06 | 2.79 |
| 30 | 836 | 51.09 | 52.13 | 53.00 | 54.04 | 55.76 | 57.65 | 59.53 | 61.25 | 62.29 | 63.17 | 64.21 | 2.82 |
| 31 | 615 | 53.01 | 54.06 | 54.94 | 55.99 | 57.73 | 59.63 | 61.54 | 63.27 | 64.32 | 65.20 | 66.25 | 2.84 |
| 32 | 311 | 54.82 | 55.89 | 56.78 | 57.84 | 59.59 | 61.51 | 63.44 | 65.19 | 66.25 | 67.14 | 68.20 | 2.87 |
| 33 | 136 | 56.53 | 57.61 | 58.51 | 59.58 | 61.35 | 63.29 | 65.23 | 67.00 | 68.07 | 68.97 | 70.04 | 2.90 |
| 34 | 50 | 58.14 | 59.22 | 60.13 | 61.21 | 63.00 | 64.96 | 66.92 | 68.71 | 69.79 | 70.70 | 71.78 | 2.93 |
| 35 | 34 | 59.63 | 60.73 | 61.64 | 62.74 | 64.54 | 66.52 | 68.50 | 70.30 | 71.39 | 72.31 | 73.40 | 2.96 |
| 36 | 11 | 61.01 | 62.12 | 63.04 | 64.14 | 65.96 | 67.96 | 69.96 | 71.78 | 72.88 | 73.81 | 74.91 | 2.98 |
| 37 | 12 | 62.27 | 63.39 | 64.32 | 65.44 | 67.27 | 69.29 | 71.31 | 73.14 | 74.26 | 75.19 | 76.31 | 3.01 |
| Total | 8034 |  |  |  |  |  |  |  |  |  |  |  |  |

**Table S6. Growth chart for estimated fetal weight (g) by percentile with SD**

| Weeks  of gestation | No of fetus (measurement) | 1^st^ | 2.5^th^ | 5^th^ | 10^th^ | 25^th^ | 50^th^ | 75^th^ | 90^th^ | 95^th^ | 97.5^th^ | 99^th^ | SD |
| --- | --- | --- | --- | --- | --- | --- | --- | --- | --- | --- | --- | --- | --- |
| 13 | 731 | 51.61 | 56.59 | 60.75 | 65.73 | 73.93 | 82.93 | 91.94 | 100.13 | 105.11 | 109.27 | 114.25 | 13.44 |
| 14 | 879 | 67.45 | 73.79 | 79.10 | 85.44 | 95.90 | 107.39 | 118.87 | 129.33 | 135.67 | 140.99 | 147.33 | 17.14 |
| 15 | 637 | 84.87 | 92.69 | 99.23 | 107.05 | 119.94 | 134.09 | 148.25 | 161.14 | 168.95 | 175.50 | 183.32 | 21.13 |
| 16 | 318 | 106.41 | 116.02 | 124.06 | 133.67 | 149.50 | 166.89 | 184.29 | 200.12 | 209.72 | 217.77 | 227.37 | 25.96 |
| 17 | 208 | 134.85 | 146.77 | 156.75 | 168.67 | 188.31 | 209.89 | 231.47 | 251.11 | 263.03 | 273.01 | 284.93 | 32.21 |
| 18 | 522 | 165.88 | 180.27 | 192.33 | 206.72 | 230.44 | 256.50 | 282.56 | 306.29 | 320.68 | 332.74 | 347.13 | 38.90 |
| 19 | 792 | 197.43 | 214.30 | 228.43 | 245.30 | 273.11 | 303.65 | 334.20 | 362.01 | 378.87 | 393.00 | 409.87 | 45.59 |
| 20 | 644 | 235.38 | 255.18 | 271.77 | 291.57 | 324.21 | 360.07 | 395.93 | 428.57 | 448.37 | 464.96 | 484.77 | 53.52 |
| 21 | 352 | 277.84 | 300.88 | 320.18 | 343.22 | 381.20 | 422.91 | 464.63 | 502.61 | 525.65 | 544.95 | 567.99 | 62.26 |
| 22 | 170 | 327.16 | 353.91 | 376.31 | 403.06 | 447.15 | 495.58 | 544.01 | 588.10 | 614.85 | 637.25 | 664.00 | 72.28 |
| 23 | 66 | 383.77 | 414.71 | 440.64 | 471.58 | 522.59 | 578.62 | 634.64 | 685.66 | 716.60 | 742.52 | 773.46 | 83.62 |
| 24 | 25 | 445.72 | 481.19 | 510.91 | 546.39 | 604.87 | 669.11 | 733.35 | 791.83 | 827.31 | 857.03 | 892.51 | 95.88 |
| 25 | 7 | 520.74 | 561.64 | 595.90 | 636.79 | 704.21 | 778.26 | 852.31 | 919.73 | 960.63 | 994.89 | 1035.78 | 110.52 |
| 26 | 4 | 595.10 | 641.31 | 680.02 | 726.22 | 802.40 | 886.06 | 969.73 | 1045.90 | 1092.11 | 1130.82 | 1177.02 | 124.88 |
| 27 | 15 | 688.53 | 741.32 | 785.56 | 838.35 | 925.39 | 1021.00 | 1116.60 | 1203.64 | 1256.44 | 1300.67 | 1353.47 | 142.69 |
| 28 | 117 | 797.79 | 858.21 | 908.82 | 969.24 | 1068.84 | 1178.24 | 1287.64 | 1387.24 | 1447.65 | 1498.27 | 1558.68 | 163.28 |
| 29 | 542 | 888.60 | 955.28 | 1011.15 | 1077.83 | 1187.76 | 1308.50 | 1429.24 | 1539.17 | 1605.85 | 1661.71 | 1728.39 | 180.21 |
| 30 | 836 | 987.72 | 1061.18 | 1122.72 | 1196.18 | 1317.28 | 1450.30 | 1583.32 | 1704.42 | 1777.88 | 1839.42 | 1912.88 | 198.53 |
| 31 | 615 | 1109.19 | 1190.88 | 1259.32 | 1341.00 | 1475.68 | 1623.60 | 1771.52 | 1906.19 | 1987.88 | 2056.32 | 2138.01 | 220.78 |
| 32 | 311 | 1236.95 | 1327.22 | 1402.85 | 1493.11 | 1641.93 | 1805.38 | 1968.84 | 2117.66 | 2207.92 | 2283.55 | 2373.82 | 243.96 |
| 33 | 136 | 1380.94 | 1480.79 | 1564.45 | 1664.29 | 1828.91 | 2009.71 | 2190.52 | 2355.13 | 2454.98 | 2538.64 | 2638.49 | 269.86 |
| 34 | 50 | 1530.06 | 1639.74 | 1731.64 | 1841.33 | 2022.16 | 2220.78 | 2419.40 | 2600.23 | 2709.92 | 2801.82 | 2911.50 | 296.45 |
| 35 | 34 | 1710.83 | 1832.33 | 1934.14 | 2055.64 | 2255.97 | 2475.99 | 2696.02 | 2896.34 | 3017.85 | 3119.65 | 3241.16 | 328.40 |
| 36 | 11 | 1882.24 | 2014.86 | 2125.98 | 2258.60 | 2477.25 | 2717.41 | 2957.57 | 3176.22 | 3308.84 | 3419.96 | 3552.58 | 358.44 |
| 37 | 12 | 2070.45 | 2215.19 | 2336.45 | 2481.19 | 2719.81 | 2981.90 | 3243.99 | 3482.61 | 3627.35 | 3748.61 | 3893.35 | 391.18 |
| Total | 8034 |  |  |  |  |  |  |  |  |  |  |  |  |
